# Characterization of *Nicotiana tabacum* genotypes possessing deletion mutations that affect potyvirus resistance and the production of trichome exudates

**DOI:** 10.1186/s12864-018-4839-y

**Published:** 2018-06-20

**Authors:** Kurtis L. Dluge, Zhongbang Song, Bingwu Wang, W. Tyler Steede, Bingguang Xiao, Yong Liu, Ralph E. Dewey

**Affiliations:** 1 Department of Crop and Soils Sciences, North Carolina State University, Raleigh, NC 27695 USA; 2 Yunnan Academy of Tobacco Agricultural Sciences, No. 33 Yuantong St., Kunming, 650021 People’s Republic of China

**Keywords:** *Nicotiana tabacum*, *VAM*, *Va*, Trichome exudate

## Abstract

**Background:**

Advances in genomics technologies are making it increasingly feasible to characterize breeding lines that carry traits of agronomic interest. Tobacco germplasm lines that carry loci designated *VAM* and *va* have been extensively investigated due to their association with potyvirus resistance (both *VAM* and *va*) and defects in leaf surface compounds originating from glandular trichomes (*VAM* only). Molecular studies and classical genetic analyses are consistent with the model that *VAM* and *va* represent deletion mutations in the same chromosomal region*.* In this study, we used RNA-seq analysis, together with emerging tobacco reference genome sequence information to characterize the genomic regions deleted in tobacco lines containing *VAM* and *va*.

**Results:**

Tobacco genotypes TI 1406 (*VAM*), K326-*va* and K326 (wild type) were analyzed using RNA-seq to generate a list of genes differentially expressed in TI 1406 and K326-*va*, versus the K326 control. Candidate genes were localized onto tobacco genome scaffolds and validated as being absent in only *VAM*, or missing in both *VAM* and *va,* through PCR analysis. These results enabled the construction of a map that predicted the relative extent of the *VAM* and *va* mutations on the distal end of chromosome 21. The RNA-seq analyses lead to the discovery that members of the cembratrienol synthase gene family are deleted in TI 1406. Transformation of TI 1406 with a cembratrienol synthase cDNA, however, did not recover the leaf chemistry phenotype. Common to both TI 1406 and K326-*va* was the absence of a gene encoding a specific isoform of a eukaryotic translation initiation factor (eiF4E1.S). Transformation experiments showed that ectopic expression of eiF4E1.S is sufficient to restore potyvirus susceptibility in plants possessing either the *va* or *VAM* mutant loci.

**Conclusions:**

We have demonstrated the feasibility of using RNA-seq and emerging whole genome sequence resources in tobacco to characterize the *VAM* and *va* deletion mutants. These results lead to the discovery of genes underlying some of the phenotypic traits associated with these historically important loci. Additionally, initial size estimations were made for the deleted regions, and dominant markers were developed that are very close to one of the deletion junctions that defines *va*.

**Electronic supplementary material:**

The online version of this article (10.1186/s12864-018-4839-y) contains supplementary material, which is available to authorized users.

## Background

For most major crop species, a vast array of useful genetic diversity is represented in germplasm collections and/or breeding lines [1]. Deciphering the unique genetic features of these lines can lead to improvements in plant quality, biotic and abiotic stress resistance, and the modification of pathways that lead to the production of novel compounds. Identification of the specific genes underlying these traits is still difficult, particularly in polypoid species such as tobacco (*N. tabacum*), which contain large, repetitive genomes and incomplete genomic resources. Recently, parsing and characterizing unique genes of interest in tobacco has become increasingly achievable with streamlined next generation sequencing protocols and the availability of additional tobacco genomic resources [2].

Tobacco Introduction (TI) 1406 contains the Virgin A Mutant (*VAM*), a well-known source of virus resistance that was originally generated through irradiation of the Virgin A cultivar [3]. Historically, its most notable attribute has been the conferral of recessive resistance toward strains of the potyviruses, PVY (potato virus Y), TEV (tobacco etch virus), and TVMV (tobacco vein mottling virus) [3–5]. Plants that possess the *VAM* mutation also carry traits that make them undesirable for cultivation, most notably certain trichome exudates are either negligible or completely missing [6]. In addition, plants containing *VAM* lack chloroplasts within the trichome head and have generally smaller or thinner leaves [7]. Deficiencies in trichome exudates have been associated with decreased resistance to the tobacco flea beetle and hornworm [6].

Glandular trichome secretions in tobacco are primarily composed of two diterpenoid groups, the macrocyclic cembranoids, α and β-cembratriene-diols (α-, β-CBT-diols), and the polycyclic labdanoids, Z-abienol and labdene-diol. Although virtually all normal tobacco plants accumulate high levels of the α- and β-CBT-diols, the Z-abienol and labdene-diol levels vary widely among germplasm lines, with these compounds typically being found in greatest amounts in tobaccos of the Oriental market type [8]. Several sucrose esters of short chain fatty acids can also be detected in tobacco leaf surface exudates, which like Z-abienol, are typically found in greatest abundance in Oriental-type tobaccos [9, 10].

Many of the genes encoding enzymes involved in the formation of tobacco trichome exudates have been characterized in *N. tabacum*. The production of CBT-diols is initiated with the cyclization of geranylgeranyl diphosphate (GGPP) into α- and β-CBT-ols by the enzyme cembratrienol synthase (CBTS). RNAi-mediated inhibition of a CBTS-encoding gene designated *CYC-1* was very effective in inhibiting the production of α- and β-CBT-ols, and α- and β-CBT-diols [11]. After the CBTS-mediated production of α- and β-CBT-ols, a cytochrome P450 enzyme encoded by the *CYP71D16* gene hydroxylates these compounds to form α- and β-CBT-diols [12, 13]. The CYP71D16-mediated reaction is very efficient, as only trace amounts of the α- and β-CBT-ols can typically be detected on the leaf surface. The biosynthesis of Z-abienol also initiates from GGPP, as a class II terpene synthase encoded by the *NtCPS2* gene converts GGPP to 8-hydroxyl-copalyl diphosphate. Subsequent production of Z-abienol from 8-hydroxyl-copalyl diphosphate is catalyzed by a kaurene synthase-like enzyme encoded by the *NtABS* gene [14]. From the analysis of over 100 diverse tobacco cultivars, it was shown that debilitating mutations in *NtCPS2* were highly associated with genotypes that lacked Z-abienol as a leaf surface compound [14].

Imaging of trichome heads from TI 1406 showed a lack of exudate buildup, though the cuticle that would normally surround the exudate appeared to be present. The most dramatic ultrastructural feature distinguishing T1 1406 trichomes, however, is the lack of chloroplasts within the head; in comparison, normal tobacco trichome heads possess numerous, well-developed chloroplasts [7]. Analysis of the leaf surface components of TI 1406 showed that this genotype produced very low levels of α- and β-CBT-diols, and trace amounts of Z-abienol and the β-methylvaleric acid-containing sucrose esters (BMVSE) [9]. Doubled haploid (DH) lines that were derived from a cross between TI 1406 and burley variety Ky14 produced a range of leaf surface chemistry phenotypes for α- and β-CBT-diols, Z-abienol and BMVSE. The observation of several DHs lines producing substantial quantities of Z-abienol and BMVSE was unexpected, given that Ky14 does not produce these compounds. The authors of this study concluded that the TI 1406 genome does in fact possess the genes capable of facilitating Z-abienol and BMVSE production, but in the presence of the *VAM* locus are unable to produce more than trace amounts of these due to the trichome secretory deficiencies caused by this mutation [9], a deficiency that they attributed to the lack of functional chloroplasts within the trichome heads of TI 1406 [7, 9].

The *va* locus originated as a single plant selection displaying a high level of potyvirus resistance that was identified from a complex F_4_ breeding population [15]. Because TI 1406 was represented within the lineage, it was presumed that the *VAM* locus was the source of the resistance observed in this plant. Interestingly, unlike plants possessing *VAM*, those with *va* possess normal trichome secretions and a normal trichome chloroplast phenotype. Since the *va* locus confers desirable virus resistance in the absence of unwanted leaf chemistry alterations, it has been widely deployed in commercial tobacco varieties. Virus resistance in TI 1406 appears to be somewhat stronger than plants carrying *va* as there are potentially two loci providing PVY resistance, each independently inherited as recessive loci *va* and *va2*. These have been proposed to restrict viral intercellular movement and restrict virus accumulation, respectively [16]. Recently, potyvirus resistance attributed to *va* was shown to be due to the absence of a specific eukaryotic translation initiation factor 4E (eIF4E) isoform [17], an isoform designated eIF4E1.S by Sierro et al. [2]. Specific eIF4E isoforms can facilitate infection, as the PVY viral genome-linked protein (VPg) mimics the 5′-cap structure of messenger RNAs, allowing it to bind to eIF4E during the process of viral replication [18]. *Va* maps to an end of linkage group 21, in a part of the chromosome that is believed to have originated from *N. tabacum*’s maternal ancestor, *N. sylvestris* [17, 19]. Although both *VAM* and *va* represent deletion mutants that confer potyvirus resistance, it is not known if the entirety of *VAM* is simply an extension of the deletion that defines *va*. It is also unknown whether *VAM* corresponds solely to the segment of chromosome 21 derived from the *N. sylvestris* progenitor, as the majority of chromosome 21 originated from the paternal *N. tomentosiformis* ancestor [19].

In the present study, we investigate the chromosomal regions and genes that are deleted in tobacco plants possessing the *VAM* and *va* loci, and examine their relationship to the virus resistance and leaf surface chemistry phenotypes. RNA-seq was used to compare tobacco lines K326, K326 containing *va* (K326-*va*), and the *VAM* carrier TI 1406 in order to identify a set of gene deletions common to both *VAM* and *va*, and those unique to *VAM*. This information was used in conjunction with recently reported draft genomes of tobacco to roughly define the location and extent of the *VAM* and *va* deletion mutations. The role of the eIF4E1.S gene in conferring potyvirus susceptibility was further characterized in transformation experiments conducted in plants possessing either the *VAM* or *va* loci. Transformation experiments were also conducted with a member of the cembratrienol synthase gene family shown to be absent in plants possessing the *VAM* locus, to test its ability to help restore trichome exudate production in these plants.

## Results

### Sequencing and transcriptome generation

RNA-seq that was performed on pooled young leaf tissue generated raw reads of 30,284,433 for K326, 58,004,150 for K326-*va*, and 61,640,362 for TI 1406. Trimming and quality control left reads of 28,309,873 for K326, 46,213,114 for K326-*va*, and 52,132,200 for TI 1406. The WT K326 reads were used initially to generate a transcriptome which was filtered of contigs with less than 10-fold coverage or 500 bp in length, resulting in a contig N50 of 1669 for 40,080 Trinity produced genes. Due to naturally occurring polymorphisms between the K326 and TI 1406 genomes, limiting false positives during alignment was crucial to keep deletion candidates to a manageable number. Therefore, K326 RNA-seq reads were used to construct the transcriptome, and the RNA-seq reads from TI 1406 were mapped against it. Another potential alignment option would have been to align all reads to each *N. tabacum* ancestral parent (*N. tomentosiformis* and *N. sylvestris*) transcriptome that are available in public databases. This may have resulted in fewer gene isoforms to parse but would have likely increased misaligned reads due to further evolutionary distance, and thus was not pursued.

### Candidate contig list creation through expression analysis

The deletion mutation that defines *va* is speculated as having been derived from the larger *VAM* deletion, of which TI 1406 was the source [15]. Therefore, contigs with dramatically lower expression in K326-*va* or TI 1406, as ranked by fold-change in comparison to K326, are candidates for being genes that are deleted in both *va* and *VAM*. Contigs exclusively lower in TI 1406 are candidates for being uniquely absent in plants with the *VAM* locus. Using default BWA-MEM settings, reads from K326, K326*-va* and TI 1406 were aligned to the K326 transcriptome we generated. Alignments were then normalized for post-processing sequencing depth, and contigs were ranked by expression fold differences between K326 and each of the other cultivars. An above zero level of TI 1406 and/or K326-*va* alignments was seen for many of the best candidate contigs on the list. The existence of a non-zero level of reads for a given contig did not disqualify it as a candidate, as this could merely be the result of a degree of cross mapping that would be expected in the highly repetitive tobacco genome.

To reduce false positives, 25,629,411 trimmed RNA-seq K326 reads (SRR955772) from Sierro et al. [2] were also aligned to our K326 transcriptome. As both sets of K326 reads come from leaf tissue grown under similar conditions, this comparison facilitated the removal of highly variable genes. Contigs with a greater than a 2-fold difference in expression between the two independent K326 sets were not considered as *va* or *VAM* candidates.

Despite the high similarity between *N. tabacum* accessions in general, false positives may arise due to inherent differences between the K326 and TI 1406 gene orthologs. K326 is a flue-cured variety while the origins of TI 1406 are not clear. An investigation of SSR markers across numerous tobacco accessions by Fricano et al. [20] showed that TI 1406 may be somewhat more related to burley rather than flue-cured varieties. To help control for potential genetic differences, a set of the burley variety TN90 leaf RNA-seq contigs (SRR1199203) were downloaded, processed with Trimmomatic and assembled into contigs using the same settings as our K326 reads. The top differentially expressed TI 1406 contigs from above were aligned with the constructed TN90 transcriptome using Blast-2.2.29+. Because TN90 contains the *va* locus, genes unique to *VAM* should be present in TN90 and absent in TI 1406. Therefore, a gene with low or no expression in TI 1406 that displayed high quality alignments between K326 and TN90 transcriptomes was viewed as further evidence that the gene was not scored as under-expressed due simply to sequence divergence. Furthermore, differentially expressed candidate contigs predicted from K326-*va* to K326 transcriptome alignments were removed if they had an excellent match in TN90.

A list of candidate genes likely to be missing in *VAM*, sorted by fold-change and created using all false positive controls, contained 55 contigs whose expression change was > 10-fold in K326 over TI 1406, or completely absent in TI 1406 (Table 1). When contigs were sorted by K326 versus K326-*va* fold change, 12 were found that displayed at least a 7-fold difference. Of these, all except contig 22586c0g2i1, whose corresponding TI 1406 to K326 fold change is 7.08, were also found within the *VAM* set in Table 1. This is consistent with the hypothesis that the entire *va* deletion was derived from *VAM*. Sequences were annotated with BLASTN when BLASTX failed to return a significant result. Among the annotated contigs in Table 1 are a heat shock protein (85964c0g1i1), a *N. tabacum* Sar8.2 gene (23466c1g2i2), and isoforms of cembratrien-ol synthase (CBTS) (33989c1g1i3). CBTS plays a major role in the production of trichome exudates and multiple isoforms, or alternative splicing variants, of this gene appear on the list.

**Table 1:** Contigs with greater than 10-fold (K326/TI 1406) or 7-fold (K326/ K326-*va*) differential expression

Contig ID	K326 coverage	K326/TI 1406	K326/K326 va	PCR	Annotation	S or T
24507c0g1i1	27.7	na	0.65	yes	Cembratrienol synthase 3	S
33989c1g1i3	408.3	2351.50	0.18	–	Cembratrienol synthase 3	S
33989c1g1i6	323.3	1443.83	0.06	yes	Cembratrienol synthase 2a	S
33989c1g1i4	204.4	1333.67	0.11	yes	Cembratrienol synthase 2a	S
33989c1g1i5	204.9	952.75	0.14	yes	Cembratrienol synthase 2a	S
33989c1g1i2	221.1	463.05	0.10	yes	Cembratrienol synthase 2a	S
8697c0g1i1	83.2	447.40	158.00	–	Uncharacterized protein LOC104224958	S
87014c0g1i1	59.5	203.87	−0.08	–	–	
23542c0g2i2	31.1	183.15	0.20	–	–	
22222c0g1i1	296.4	127.47	29.46	–	Auxin-repressed 12.5 kDa protein-like	S
33989c1g2i1	141.4	113.38	0.36	yes	Cembratrienol synthase 2a	S
22534c0g3i1	21.2	79.41	29.55	yes*	Uncharacterized protein LOC104232912	S
35943c0g2i1	24.3	48.72	35.73	yes*	3-Isopropylmalate dehydratase	S
23466c1g2i1	656.1	47.47	−0.39	yes	Sar8.2c	T
2175c0g2i1	55.9	42.48	24.70	–	Trehalose-phosphate phosphatase G	S
22475c0g1i2	56.0	36.96	18.54	yes*	Cyclic phosphodiesterase-like	S
2175c0g1i1	56.6	33.51	22.45	–	Trehalose-phosphate phosphatase G	S
33215c0g1i1	75.0	33.11	0.18	–	Uncharacterized protein LOC107810047	
64746c0g1i1	22.7	32.65	327.11	–	–	
28889c6g4i1	34.6	30.27	0.40	–	Uncharacterized protein LOC107777218	T
42740c0g1i1	40.8	25.89	0.24	yes	Uncharacterized protein LOC107765064	T
27218c1g1i1	10.3	25.31	0.30	–	Uncharacterized protein LOC107786237	S
32651c2g7i3	10.8	25.24	−0.18	–	F-box protein	T
65564c0g1i1	29.0	23.74	0.05	–	–	
23466c1g2i2	966.4	23.70	−0.36	yes	Sar8.2 k	T
25192c0g1i1	34.1	22.46	0.34	–	Bifunctional purple acid phosphatase 26	S
25436c0g1i2	19.4	22.33	0.22	yes	Uncharacterized protein LOC104238501	S
8352c0g1i1	10.9	21.95	0.79	–	Uncharacterized protein LOC107812316	T
25661c1g1i3	42.9	19.09	11.64	yes*	Cyclic phosphodiesterase	S
40439c0g1i1	10.7	18.95	0.19	–	–	
22779c0g1i1	118.1	18.95	17.29	–	heat- and acid-stable phosphoprotein	S
86871c0g1i1	18.4	17.78	0.73	yes*	Uncharacterized protein LOC104224418	S
20225c0g1i1	28.8	17.76	0.18	yes	–	
22899c0g2i1	53.5	17.74	0.28	–	Uncharacterized protein LOC107760627	T
21258c0g1i2	61.9	17.72	0.02	–	Nucleoside diphosphate kinase 3	S
75249c0g1i1	44.0	16.17	−0.23	–	Uncharacterized protein LOC107794475	S
18187c0g2i2	17.8	15.68	0.11	yes	Uncharacterized protein LOC104232736	S
64896c0g1i1	10.3	14.35	0.36	–	–	
26833c0g2i1	24.0	14.24	11.16	–	Ethylene-responsive transcript. Factor 4	S
16240c0g1i1	26.9	14.05	0.05	yes	4-coumarate--CoA ligase	S
25958c0g1i2	17.8	12.81	7.69	yes*	Heat stress transcription factor A	S
65623c0g1i1	24.4	12.78	0.59	–	–	
19615c0g1i2	10.6	12.48	0.58	–	Interaptin	T
3660c0g2i1	10.3	12.47	−0.02	–	Diaminopimelate epimerase,	S
30135c0g1i1	15.0	12.36	1.03	–	Cytochrome P450 78A5	S
3660c0g1i1	11.8	12.26	0.10	–	Diaminopimelate epimerase,	S
30979c0g1i5	50.1	12.23	1.19	–	GDSL esterase/lipase	S
33333c2g1i1	18.7	11.97	−0.05	–	–	
12543c0g1i1	35.3	11.85	0.28	–	Uncharacterized protein LOC104210975	S
28570c0g1i1	42.9	11.57	0.04	yes	Protein disulfide-isomerase	S
85964c0g1i1	10.5	11.00	0.20	yes*	Heat shock protein 83	S
23580c0g1i1	104.5	11.00	−0.33	yes	Early light-induced protein 1, chloroplastic	S
18777c2g1i1	11.4	10.71	0.56	–	Uncharacterized protein LOC107823745	S
26611c0g1i2	29.2	10.55	−0.18	yes	–	
32166c0g3i1	25.4	10.45	0.37	–	Uncharacterized protein LOC107771105	T

Columns 3 and 4 are expressed in terms of expression fold change. Column 5 shows contigs which failed to amplify using PCR in TI 1406; those that also failed to amplify in K326-*va* and TN90 are indicated with an asterisk. Column 7 shows the best matching ancestral tobacco species (S = *N. sylvestris,* T = *N. tomentosiformis*)

There are two notable omissions for the genes listed in the Table 1. First, contig 30706c0g2i3, corresponding to the eIF4E1.S isoform originating from the *N. sylvestris* genome and implicated in PVY susceptibility [17] is absent. Contig 30706c0g2i3 has an expression fold change of 1.95 lower in TI 1406 and 1.33 lower in K326-*va*. As there are numerous eIF4E isoforms in the tobacco genome that share high sequence identity, legitimate expression differences were likely masked for 30706c0g2i3 due to cross-mapping during alignment. Second, contigs annotated as light-harvesting chlorophyll a/b-binding proteins (lhcb), some of which have been reported as missing in *va* [17] (and therefore likely *VAM* as well), encounter the same issue. The lhcb contigs that show the greatest similarity to S genome lhcb genes appear with only minimally higher expression in K326 than K326-*va* or TI 1406. Both S genome lhcbs and 30706c0g2i3 show greater differential expression when processed RNA-seq reads are aligned with no mismatches or gaps allowed. However, we found imposing this level of stringency on gross alignments was less useful, as false positives arising from the natural polymorphisms that exist between K326 and TI 1406 became overwhelming.

As *N. tabacum* originally arose through an ancient hybridization between individuals most closely related to *N. sylvestris* and *N. tomentosiformis* [21] and each of these species has been sequenced in some depth [22], potential information about genetic ancestry can be gained by looking at which of these two ancestral tobacco species is a better match for a given differentially expressed contig. Provided adequate coverage and annotations exist, higher quality alignments are expected with the *N. sylvestris* genome for genes potentially missing in *va* and *VAM* plants. This is due to: (1) *N. sylvestris* being highly susceptible to potyvirus infection while *N. tomentosiformis* is resistant; (2) extra-cuticular CBT-diols are present on the leaves of *N. sylvestris* and are not found in *N. tomentosiformis* [23]; and (3) the *va* locus has be previously mapped to a portion of chromosome 21 expected to originally have been inherited from *N. sylvestris* [17]. Thirty-five of the contigs shown on Table 1 returned *N. sylvestris* as the top alignment, while nine contigs matched most closely to *N. tomentosiformis* sequences.

### Candidate sequence validation through PCR and southern blot analysis

We attempted to design primers to amplify each of the contigs found in Table 1 and all known eIF4E isoforms, including the suspected PVY susceptibility isoform eIF4E1.S (30706c0g2i3) (Additional file 1: Table S1). Primers were checked against the complete list of our K326 contigs and online resources in an effort to improve their specificity. Primers were run with K326, K326-*va*, and TI 1406 genomic DNA isolated from the plants used in RNA-seq analysis. Additionally, DNA from TN90 was included as it also possesses the *va* locus.

**Fig. 1:**
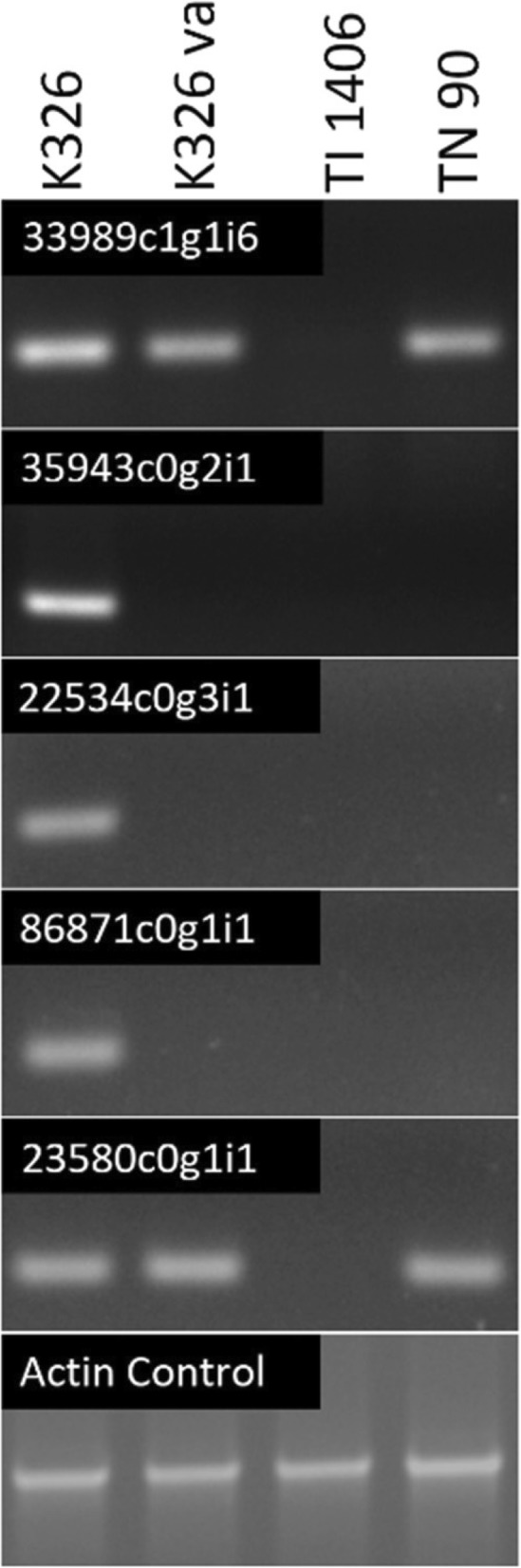
PCR validation of genes representing the two classes of contigs revealed by RNA-seq analysis. PCR analysis of contigs 35943c0g2i1, 22534c0g3i1 and 86871c0g1i1 shows the typical pattern of genes that are absent in lines containing either *va* (K326-va and TN 90) or *VAM* (TI 1406). Contigs that only fail to amplify in plants with the *VAM* mutation are exemplified by 33989c1g1i6 (CBTS-2a) and 23580c0g1i1

**Fig. 2:**
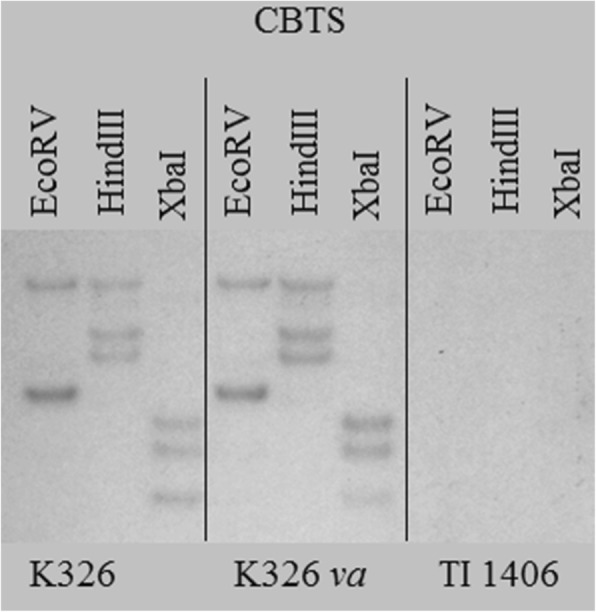
Southern blot analysis using the *NtCBTS-2a* cDNA as hybridization probe. Genomic DNAs from tobacco lines K326, K326-*va* and TI 1406 were digested with EcoRV, HindIII, and XbaI and analyzed on the same gel

Twenty-two of the contigs listed in Table 1 failed to amplify in TI 1406, seven of which also failed to amplify with genomic DNAs isolated from K326-*va* and TN90. Although the other 33 contigs could not be validated by PCR, they are still considered potential candidates since it was their short size, repetitive nature and/or the inability to produce discriminating primers that prevented definitive PCR testing. Representative examples of the PCR analyses are shown in Fig. 1. Included among the contigs that were PCR verified are a 3-isopropylmalate dehydratase (35943c0g2i1), a gene that was also identified in the RNA-seq analysis of the *va* locus reported by Julio et al. [17], and several contigs encoding CBTS genes (represented by 24507c0g1i1, 22989c1g1i3, 33989c1g2i1, 33989c1g1i2, 33989c1g1i4, 33989c1g1i5, and 33989c1g1i6). The multiple entries annotated as CBTS-2a or CBTS-3 may be due to incomplete or differently spliced de novo contig assemblies. In *N. sylvestris*, three CBTS genes, *NsCBTS-2a*, *NsCBTS-2b*, *NsCBTS-3*, and one likely pseudogene, CBTS-4, have been reported [24]. BLAST was used to search for additional contigs with similarity to *N. sylvestris* CBTS sequences within our assembled K326 transcriptome. All potential isoforms revealed in these searches, however, were those already represented in Table 1. To directly test for the presence of CBTS-homologous genes in TI 1406, an amplified portion of the CBTS-2a cDNA was used as a hybridization probe in a Southern blot analysis. The blot shown in Fig. 2 shows that all bands are completely missing in TI 1406, suggesting that all CBTS-like genes are absent in the *VAM* mutant line.

### Deletion size estimation through analysis of public genome data

**Fig. 3:**
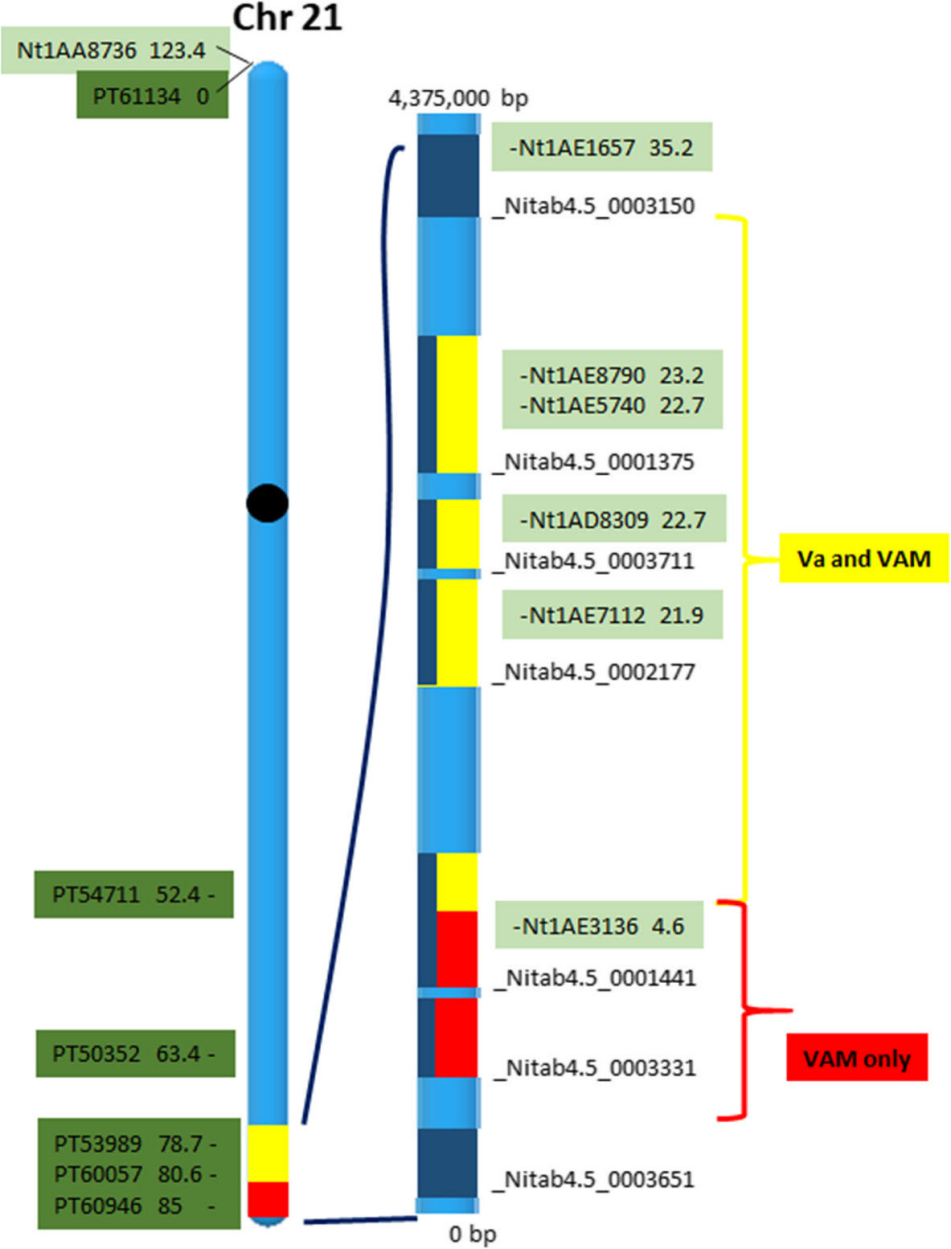
Map of tobacco chromosome 21, highlighting the region encompassing the *VAM* and *va* loci. Anchored scaffolds missing in both *VAM* and *va* plants are shown in yellow and those uniquely missing in *VAM* in red. Infinium markers used by Edwards et al. [25] and aligned to scaffolds are in light green along with their relative distance in cM. Markers described by Bindler et al. [13] are dark green along with their predicted distance in cM. Note that the two marker systems begin at different ends of the chromosome and differ substantially in overall predicted map distance

The RNA-seq results support the hypothesis that the *VAM* locus is a deletion mutant encompassing a larger proportion of chromosome 21 than the deletion that defines *va*. To gain insight into the physical sizes of the respective deletions, BLAST was used to align contigs with significant K326/TI 1406 expression changes (> 3-fold) to scaffolds of the v4.5 K326 tobacco genome deposited in GenBank by Edwards et al. [25]. Alignments were individually inspected, as contigs representing mRNAs may not be entirely contained in a single DNA scaffold or may extend over multiple exons. Nevertheless, a ≥ 99% similarity was required over aligned sections. Fig. 3 displays a representation of the layout of *va/VAM* within an expanded section of the end of the long arm of chromosome 21, shown with scaffolds that have been anchored on the chromosome by Edwards et al. [25]. Each of the anchored scaffolds in the region predicted to be encompassed by *VAM* were further validated using PCR (data not shown). Additional steps were taken to fill in gaps between anchored scaffolds. First, Tobacco Infinium-30 k markers (https://solgenomics.net/cview/ map.pl?map_version_id = 178) were aligned to all Edwards et al. [25] scaffolds. Positive hits to anchored scaffolds are shown highlighted in light green in Fig. 3. Scaffolds containing a marker that mapped in the expected *VAM* deletion region, but have not been anchored to the physical map are shown in Table 2. Finally, a draft genome assembly generated by the Yunnan Academy of Tobacco Agricultural Sciences for tobacco cultivar RBST was aligned to Edwards et al. [25] scaffolds and added to our VAM assembly to bridge gaps in the anchored assembly. These scaffolds, in predicted order, are shown in Table 2 along with their length and the number of genes predicted to be present on the scaffold (v4.5 genome annotations [25]), and whether highly differentially expressed genes from our study were found on the scaffold.

**Table 2:** Predicted VAM scaffolds sorted by a hybrid physical/genetic mapping order

Anchored Scaffolds	Scaffolds with markers	RBST ordered Scaffolds	Scaffold length	Marker ID	Map cM	Diff-gene	# of genes
Nitab4.5_0003331			322,448	–	–	–	15
	Nitab4.5_0007068		139,279	Nt1AC8849 Nt1AE0363	0 2.3	Y*	8
	Nitab4.5_0008902		97,203	Nt1AC4113	1.8	–	3
	Nitab4.5_0013033		33,607	Nt1AC7974	2	Y	2
	Nitab4.5_0008971		95,815	Nt1AD4189	2.3	Y	6
Nitab4.5_0001441	Nitab4.5_0001441	Nitab4.5_0001441	550,039	Nt1AE3136^a^	4.6	Y*	15
		Nitab4.5_0003268	320,528	–	–	Y*	12
		Nitab4.5_0010227	72,084	–	–	Y	1
		Nitab4.5_0006402	160,683	–	–	Y	1
		Nitab4.5_0002210	421,245	–	–	Y	13
		Nitab4.5_0002814	355,923	–	–	Y	16
		Nitab4.5_0002507	388,355	–	–	Y*	13
		Nitab4.5_0002458	393,533	–	–	Y*	13
		Nitab4.5_0005928	177,860	–	–	–	2
		Nitab4.5_0004518	237,847	–	–	–	1
		Nitab4.5_0006329	163,221	–	–	–	1
		Nitab4.5_0001037	657,976	–	–	–	2
		Nitab4.5_0008217	112,516	–	–	–	1
	Nitab4.5_0003843	Nitab4.5_0003843	276,436	Nt1AC3736	20.4	–	6
Nitab4.5_0002177	Nitab4.5_0002177	Nitab4.5_0002177	424,490	Nt1AE7112^a^	21.9	Y*	17
Nitab4.5_0003711	Nitab4.5_0003711	Nitab4.5_0003711	284,557	Nt1AD8309	22.7	Y	7
Nitab4.5_0001375	Nitab4.5_0001375		566,801	Nt1AE5740 Nt1AE8790	22.7 23.2	Y*	18
	Nitab4.5_0003487		300,957	Nt1AA4543	32.3	Y*	11

^a^Markers did not have a perfect match to scaffold due to incomplete scaffold sequencing

*Diff-gene*: a Y indicates that at least one of our contigs displaying > 3-fold K326/TI 1406 expression change aligned to this scaffold; a Y* means a contig from Table 1 appears on the scaffold

**#** *of genes*: genes predicted by Edwards et al. [25] to be within this scaffold (annotations found in Additional file 2)

Two *N. tabacum* SSR markers located at the end of chromosome 21 developed by Bindler et al. [19], designated PT60946 and PT60057, are reported as being absent in *va* plants [17]*.* PT60946 and PT60057 along with other markers from [19] in the same region of chromosome 21 were tested in lines K326, K326-*va*, TN90 and TI 1406. In agreement with the findings of Julio et al. [17], PT60946 and PT60057 did not amplify in K326-*va* or TN90. As expected, PT60946 and PT60057 also did not amplify genomic DNA from TI 1406, whereas all other tested markers in this region were PCR positive in all lines. Using the markers in Table 2 and the presence of genes with significant K326/TI 1406 expression change, all scaffolds between Nitab4.5_0003331 and Nitab4.5_0003487 have the potential to be missing in *VAM*. The markers shown represent a map distance of 32.3 cm (Tobacco Infinium-30 k). Scaffolds shown above Nitab4.5_0001441 in Table 2 are predicted to be deleted only in *VAM*, and not *va*.

Of particular note, Nitab4.5_0002814 and Nitab4.5_0002210 have multiple chlorophyll a-b binding proteins matching those found to be missing in plants with *va* according to Julio et al. [17]. Nitab4.5_0002814 contains eIF4E1.S and Nitab4.5_0002210 contains the PCR verified contigs S27183 and S05079 from [17]. Two other PCR verified genes from their study, S25284 and S14740, likely reside on Nitab4.5_0001375. The CBTS gene family seems to be poorly assembled in current genomic resources, with the 1453 bp-long Nitab4.5_0221271 being the closest match for CBTS-2a, and no other high quality scaffold hits for other CBTS genes are found in publically available databases.

The total scaffold length for the v4.5 *N. tabacum* genome scaffolds shown in Table 2 is 6.55Mbp, where ~1Mbp are exclusively missing in *VAM* and ~ 5.5Mbp are absent in both *VAM* and *va*. Based on the v4.5 genome annotations, the scaffolds in Table 2 are predicted to contain 184 genes. Individual gene annotations are displayed in (Additional file 2: Table S2). The K326 genome scaffolds deposited in GenBank by Sierro et al. [2] (assembly: GCA_000715075.1) were also investigated for their potential to expand our knowledge of *VAM,* but were ultimately not included because they did not substantially add to or alter the model of *VAM* and *va* structure shown in Fig. 3 and Table 2.

### Localizing a *va* end point.

**Fig. 4:**
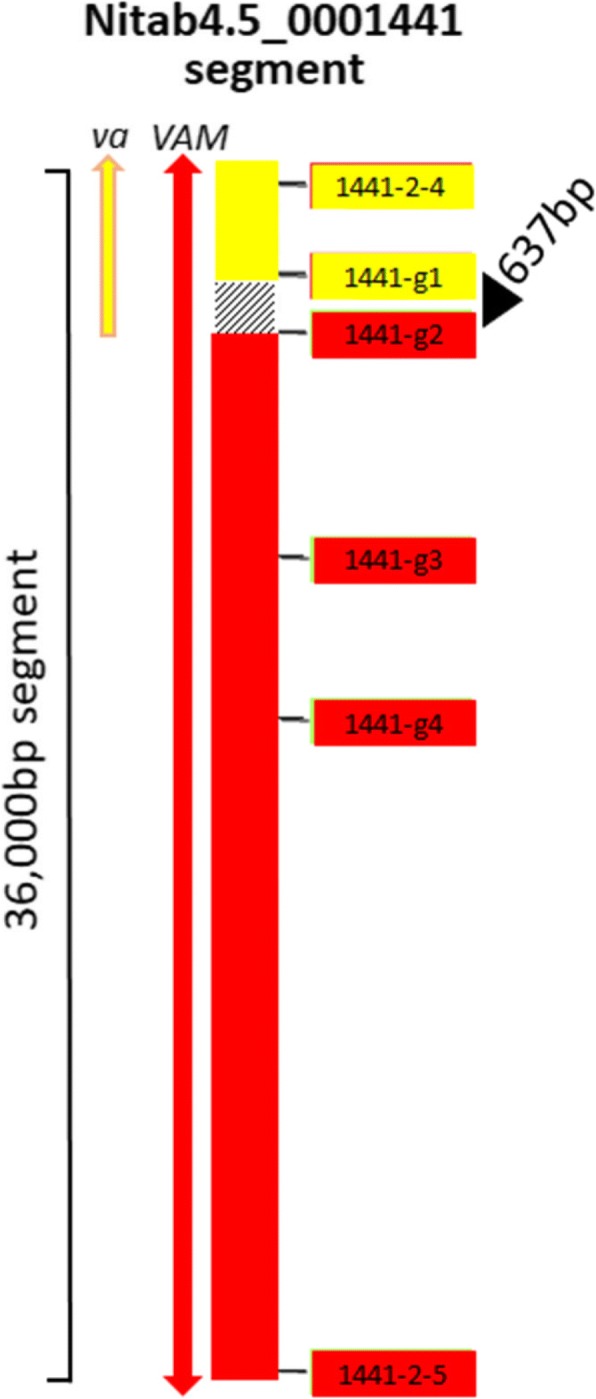
A 36,000 bp region of scaffold Nitab4.5_0001441 including a proposed *va* deletion junction. Yellow and red highlighted numbers represent PCR markers used to localize one end of the *va* deletion region. Markers highlighted in red score positive in both wild type plants and plants with *va*, and are negative in TI 1406; markers shown in yellow fail to amplify a product in plants containing either *va* or *VAM*. The 637 bp region separating markers 1441-g1 and 1441-g2 (not drawn to scale) is highlighted

If one could identify an exact deletion junction for the *va* locus, it should be possible to create co-dominant markers to assist in the breeding of this important recessive locus into additional tobacco varieties. As an attempt to define such a junction, primers were designed along the length of the anchored genomic scaffolds expected to be absent or at least partially missing in *va* (primers listed in Additional file 1: Table S1). PCR results predicted that the terminal end of *va* lies within scaffold Nitab4.5_0001441 (Fig. 4). Alignments to the pre-existing TN90 tobacco genomic assembly (Sierro et al. [2]) failed to show the exact *va* end point due to a lack of coverage in the particular area of interest. By generating and testing a series of additional PCR-based markers generated across against Nitab4.5_0001441, we were able to localize the likely *va* end point to a 637 bp region (nucleotides 306,892–307,529 of Nitab4.5_0001441). Although 637 bp is a short enough distance for determining the exact deletion junction through genome walking, multiple attempts to walk through the area were only successful in the K326 control line; no legitimate genome walking products were obtained from genomic DNAs isolated from either TN90 or K326-*va* (data not shown).

### Transformation of K326*-va* and TI 1406 with eIF4E1.S

**Fig. 5:**
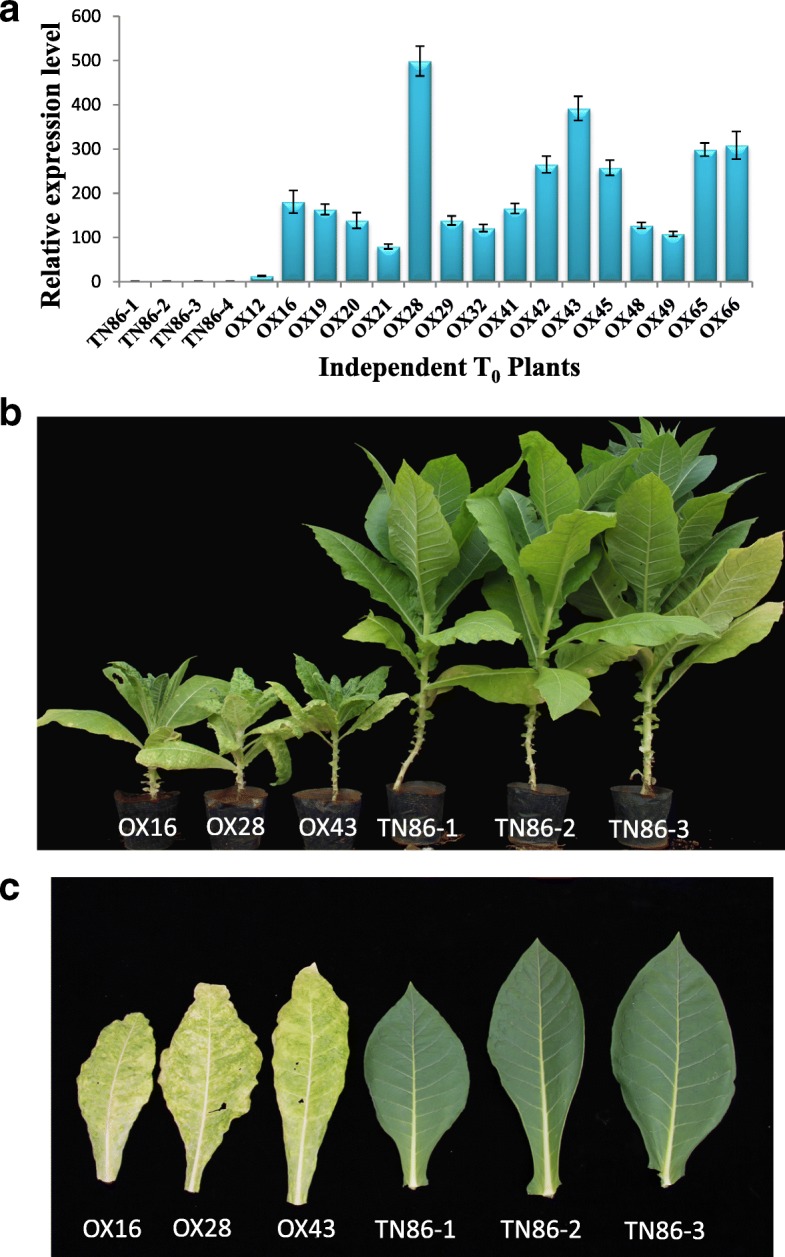
Expression of eIF4E1.S in TN86 restores susceptibility to PVY. **a** Relative expression of *eIF4E1.S* (in comparison to an endogenous actin gene) of four vector control and 16 35S::eIF4E1.S T_0_ transgenic plants using qPCR. Whole plant (**b**) and individual leaf (**c**) phenotypes from three independent T_0_ 35S::eIF4E1.S (OX) and vector control (TN86) plants are shown 21 days post-infection with strain PVY^ZT-5^

Two previous reports have shown that the absence of the single, specific eIF4E1.S isoform of the large eIF4E gene family is highly correlated with PVY susceptibility in tobacco [2, 17]. The most compelling evidence that the lack of eIF4E1.S function is causally responsible for the PVY resistance phenotype in plants possessing *va* (as opposed to other genes located within the deleted chromosomal region) comes from the observation by Julio et al. [17] that tobacco plants carrying an ethylmethane sulfonate-induced premature stop codon in eIF4E1.S showed a PVY resistance phenotype similar to plants with *va*. If the lack of a functional eIF4E1.S gene is indeed responsible for resistance to potyvirus infection, then it would be predicted that the genetic complementation of tobacco lines possessing *va* or *VAM* with a normal copy of the gene would restore a susceptibility phenotype. To test this, a wild type eIF4E1.S cDNA was cloned into a plant expression vector under the transcriptional control of the CaMV 35S promoter and introduced into TN86, which was the original tobacco variety developed containing the *va* gene [15]. As show in Fig. 5a, quantitative RT-PCR analysis revealed several T_0_ transgenic events in which eIF4E1.S was successfully expressed. Inoculation of the eIF4E1.S-transformed TN86 plants with PVY^ZT-5^, an isolate of PVY found in Southern China, resulted in a chlorotic mosaic leaf phenotype and stunted growth. In contrast, TN86 plants transformed with the control vector showed no symptoms of virus infection. Examples of each genotype 21 days post-inoculation are shown in Fig. 5b and c. Biochemical evidence of a robust PVY infection in the non-inoculated leaves of the eIF4E1.S-expressing plants was obtained by ELISA and Immunostrip assays using antibodies directed against the PVY coat protein (Additional file 3: Table S3).

In addition to TN86, 35S::eIF4E1.S constructs were introduced into TI 1406 and K326*-va*. Some T_0_ individuals were inoculated with TEV, and others were inoculated with a particularly necrotic strain of PVY (PVY^NN^). Similar to the results obtained in the TN86 background, symptoms of virus infection were readily observed in TI 1406 and K326-*va* plants transformed with the eIF4E1.S transgene and not in vector only controls (Additional file 4: Figure S1). These results support the conclusion that the re-introduction of eiF4E1.S gene into tobacco plants containing the *va* or *VAM* deletion mutations is sufficient for restoring potyvirus susceptibility to these plants.

### Investigation of genes involved in diterpene synthesis in TI 1406

As described in Background, previous studies of TI 1406 have concluded that the failure of plants possessing the *VAM* mutation to produce leaf surface diterpenes (as well as the sucrose ester BMVSE) is due to a lack of functional chloroplasts in the heads of glandular trichomes in these plants [7, 9, 26], as opposed to the alternative model that the genomes of these plants simply fail to have the genes/enzymes required to synthesize these compounds. This is best exemplified by the study by Nielson and Severson [9] which showed that the genes needed for Z-abienol and BMVSE production were indeed present in the TI 1406 genome, but could not be functionally manifest unless separated from the trichome deficiency-inducing *VAM* locus (called *te* in that paper). One of the most striking results of our RNA-seq (and Southern blot) analysis of TI 1406 was the absence of CBTS genes in this line (Table 1; Fig. 2). Within the context of the current model of *VAM* locus function, there are two possible explanations of the relationship between the lack of CBTS genes in TI 1406 and its defective trichome secretory phenotype. First, the production of α-and β-CBT-diols via CBTS activity may be required for the formation of functional chloroplasts in the heads of secretory trichomes in tobacco plants. Given the presumption that a functional chloroplast may first need to exist in order to enable production of the necessary GGPP substrate for the CBTS catalyzed reaction, this scenario arguably has a low likelihood; nevertheless, because of the observed loss of this entire gene family in tobacco plants possessing *VAM,* this possibility should be given some consideration. Second, the restoration of CBTS gene activity in *VAM* plants alone is not sufficient for enabling the synthesis of these compounds, as another gene(s) within the deletion region is responsible for the trichome-specific chloroplast developmental defect. In the first scenario the restoration of CBTS gene activity would be predicted to complement the trichome secretory defects of TI 1406 plants, whereas in the second scenario it would not, and α-and β-CBT-diol production would still not be observed even after reintroduction of a functional CBTS gene.

As an initial step in exploring this phenomenon, a comparison was made between expression levels of the genes unique to CBT-diol and Z-abienol production by aligning the RNA-seq reads from K326 and TI 1406 to the assembled K326 transcriptome. *CYP71D16* encodes a cytochrome P450 that is expressed specifically in glandular trichomes and functions after the CBTS step by catalyzing the conversion of CBT-ols to CBT-diols [13]. *NtCPS2*, encoding a class-II terpene synthase, and *NtABS*, encoding a kaurene synthase-like protein, are the structural genes responsible for the production of Z-abienol from GGPP [14]. Expression differences between K326, K326-*va* and TI 1406 for this group of diterpene-associated genes are shown in Table 3. Among these, only contigs corresponding to CBTS appear to be differentially expressed. Furthermore, visual inspection of the alignments of the reads corresponding to *NtCPS2, NtABS,* and *CYP71D16* from TI 1406 revealed no polymorphisms that would suggest that the encoded enzymes would not be functional (data not shown). These results are consistent with the hypothesis proposed by Nielson and Severson that TI 1406 individuals possess a functional Z-abienol pathway, despite its lack of production in these plants [9]. We also conclude that the *CYP71D16* gene that is responsible for conversion of α- and β-CBT-ols to α -and β-CBT-diols is also fully functional.

**Table 3:** Select tobacco diterpenoid pathway genes

Gene	Best contig match^a^	K326/K326-va fold change	K326/TI 1406 fold change
*CYP71D16*	32566c0g1i2	−0.09	0.68
*NtCPS2*	20163c0g2i1	0.34	0.14
*NtABS*	34936c0g2i11	0.02	−0.15
*NtCBTS-2a*	33989c1g1i6	0.06	1443.83
*NtCBTS-3*	33989c1g1i3	0.18	2351.5

^a^The contig from our K326 transcriptome with the best match with sequences entered in GenBank

To test whether the expression of CBTS activity in TI 1406 is capable of restoring the synthesis of α- and β-CBT-diols in this background, a full length version of *NtCBTS-2a* was PCR amplified from K326 using primers (cembBam2_F and cembSac2_R, Additional file 2: Table S2) designed based on the *NsCBTS-2a* sequence of *N. sylvestris* [24]. BLASTN alignments confirmed that *NtCBTS-2a* is the same CBTS gene as the one designated *CYC-1* by Wang and Wagner [11] who generated RNAi constructs against its sequence to demonstrate that down regulation of this gene effectively inhibited α- and β-CBT-diol production in tobacco. Two promoters were used to drive expression of *NtCBTS-2a*, the 35S promoter of CaMV and the native *NtCBTS-2a* promoter (designated CEMBpro). The latter promoter sequence was obtained by amplifying a 1812 bp region upstream of the *NtCBTS-2a* initiation of translation site. Positions − 1 through − 988 (in relation to the start ATG) of this 1812 bp sequence are > 99% identical to the 988 bp region of the *Nicotiana sylvestris NsCBTS-2a* gene promoter that was previously shown to confer a high level of trichome-specific gene expression (Genbank ID HM241147.1) [24].

**Fig. 6:**
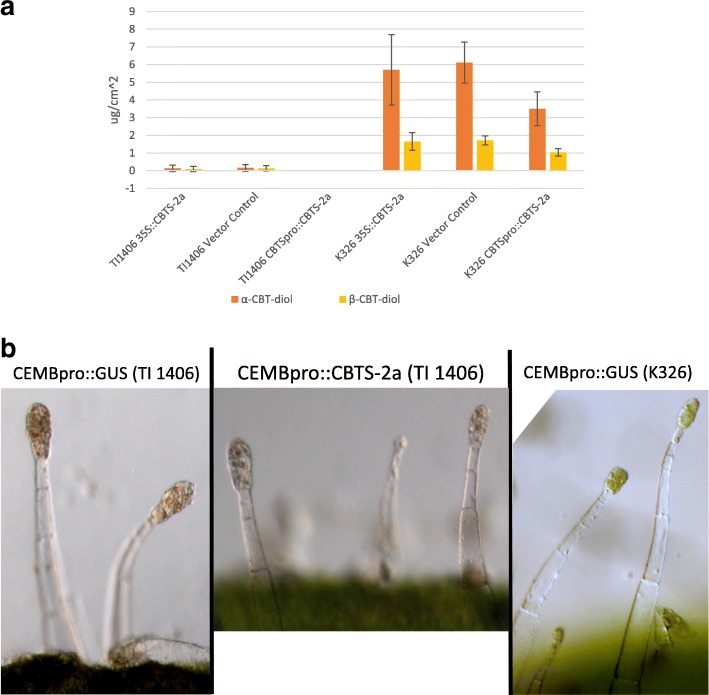
Ectopic expression of *NtCBTS-2a* fails to restore the ability of TI 1406 trichomes to produce α-and β-CBT-diols. **a** α-and β-CBT-diol content of TI 1406 plants transformed with 35S::CBTS-2a (*n* = 15), Vector Control (*n* = 10) and CEMBpro::CBST-2a (n = 15); and K326 plants transformed with 35::CBTS-2a (n = 10), Vector Control (n = 10) or CEMBpro::CBTS-2a (n = 10). Confidence intervals (95%) are provided. **b** Light microscopy images of transformed K326 and TI 1406 trichomes showing lack of chloroplast recovery in TI 1406 with CBTS-2a

A minimum of 10 independent T_0_ individuals were generated using 35S::CBTS-2a, CEMBpro::CBTS-2a and vector-only constructs in both TI 1406 and K326 backgrounds. Chemical analysis of leaf surface washes of all T_0_ plants generated in this study failed to reveal a single TI 1406 plant that accumulated any more than negligible amounts of α- and β-CBT-diols, regardless of the promoter used. These results, combined across all T_0_ individuals in each genotypic class are shown in Fig. 6a. In contrast, all transgenic plants in this study in the K326 background showed substantial α- and β-CBT-diol accumulation on their leaf surfaces. Interestingly, the introduction of 35S::CBTS-2a and CEMBpro::CBTS-2a constructs in K326 did not lead to α- and β-CBT-diol accumulation levels that were significantly different from that observed in the vector-only controls (Fig. 6a), suggesting that the endogenous CBTS activity in K326 is already quantitatively metabolizing the available GGPP substrate. Given that the reintroduction of *NtCBTS-2a* in TI 1406 did not alter the leaf surface chemistry phenotype of the plant, it was not surprising that the chloroplast-deficient phenotype of the trichome heads also remained unchanged (Fig. 6b).

## Discussion

### Nature and composition of *va* and *VAM*

In this study, we investigated the chromosomal regions associated with the *va* and *VAM* deletion mutations. Previous studies have shed light on certain specific genes exclusive to *va* and their location within the tobacco genome [2, 17]. However, the magnitude of the deletion that defines *va,* as well as its relationship to the larger *VAM* deletion from which *va* was believed to have been derived were unknown. Alignment of RNA-seq reads to a de novo assembled K326 transcriptome led to the identification of 55 contigs whose expression levels were at least 10-fold lower in TI 1406 (Table 1).

In creating this differentially expressed contig list, steps were taken to bring it to a high standard. The high potential for false positives, due to sequence or sequencing differences in homologous contigs of K326 versus TI 1406 was reduced by utilizing publically available K326 RNA-seq data to identify and remove contigs with altered expression that may have been independent of the *va* or *VAM* loci. Additionally, requiring that the contigs have close sequence similarity between our K326 transcriptome and an assembled TN90 transcriptome reduced the impact of inherent TI 1406/K326 differences. As K326*-va* and K326 are near-isogenic lines and the origin of TI 1406 is not clear, there will inevitably be more false positives in the K326/TI 1406 expression comparisons. Twenty-two contigs from Table 1 were validated by PCR as not being able to be detected in tobacco genomes containing the *VAM* or *va* mutations, 15 of which were exclusively missing in *VAM*. Due to our inability to develop suitable PCR primers that could specifically discriminate the remaining 33 contigs of Table 1, we are unable to conclude which of these may also be represented within the chromosomal regions deleted in *VAM/va* as opposed to being localized elsewhere in the genome and being differentially expressed as a secondary consequence of the deletion on chromosome 21.

Through aligning markers and genes from Table 1 to publicly available tobacco genome scaffolds and later amplifying targeted regions of these scaffolds, we were able to define a set of anchored and unanchored scaffolds which are likely to be missing in *va* and/or *VAM* plants. Additional genome assembly data developed at the Yunnan Academy of Tobacco Agricultural Sciences was used to define the order of numerous unanchored scaffolds to ones that have been anchored (Table 2). Insights into scaffold order were also gained by identifying unanchored scaffolds that contained previously published markers, either SSR [19] or Tobacco Infinium-30 k [25], which had been mapped to this region of chromosome 21. From these collective analyses, the *VAM* deletion is estimated to encompass a minimum of 24 scaffolds representing 6.55 Mbp and 184 genes. Based on the markers and map distances calculated, *VAM* would represent a deleted area encompassing at least 32.3 cM (of a 123.4 cM total of chromosome 21 estimated using Tobacco Infinium-30 k markers). The predicted *va* deletion spans the majority of that represented by *VAM*, including 18 scaffolds over a 5.5 Mbp region containing 143 genes with a genetic distance of ~ 27.7 cM.

Scaffold Nitab4.5_0003651 is the last anchored scaffold on the *VAM*-containing long arm of chromosome 21 (Fig. 3). Genes on this scaffold showed slightly higher expression in K326 than TI 1406. Attempts to PCR validate sections of the scaffold resulted in amplification in all backgrounds. However, as the genes in this area may be repetitive, masking expression differences and the hindering the ability to design differential PCR primers, we cannot eliminate the possibility that the *VAM* deletion extends all the way to the end of the chromosome.

As a caveat to the proposed structure and content of the *VAM* and *va* deletion mutations presented here, we acknowledge that they were generated under the assumption that the events leading to creation of *VAM* and *va* resulted in “clean” deletions, whereby a large contiguous chromosomal region of chromosome 21 was removed. It is entirely possible that these deletion mutations are more complex, representing the elimination of certain portions of the native chromosome within this region while retaining others, possibly in rearranged form or order. Thus, the structures and data presented in Fig. 3 and Table 2 should be viewed as a working model, with the understanding that the true nature of *va* and *VAM* will only be fully understood by whole genome sequence analysis of lines possessing these loci.

Whole genome sequence information would also be useful in identifying specific breakpoints at the two deletion junctions (and possibly more if the nature of the deletion is complex) in wild type versus *va* individuals. The *va* locus has been introduced into several commercial tobacco varieties as a valuable source of potyvirus resistance. The transfer of *va* to new cultivars, however, is not simple due to its recessive phenotype and the fact that all molecular markers directed toward this locus to date are dominant, and thus cannot distinguish homozygous dominant individuals (*VA/VA*) from heterozygous carriers (*VA/va*) in segregating populations. Knowledge of the sequence spanning a deletion break in genomes with *va* could enable the development of PCR-based markers capable of uniquely amplify *va*, which when coupled with primers corresponding to the wild type sequence at the breakpoint junction could be used as co-dominant markers and greatly facilitate the breeding of this important trait into new varieties. Despite our numerous attempts at genome walking, however, we were unable to identify the location of the deletion breakpoint within a 637 bp region of scaffold Nitab4.5_0001441.

### Exploration of CBTS and trichome phenotypes in *VAM*

Trichomes of TI 1406 individuals lack chloroplasts, and instead possess unusual structures described as membrane-bound or free inclusions [7]. Our results suggest that genes involved in the *cis*-abienol pathway and the *CYP71D16* gene of the CBT-diol pathway are expressed at normal amounts in TI 1406. Furthermore, the sequences of these genes in TI 1406 possess no mutations, which strongly suggests that these steps of in diterpene synthesis in TI 1406 are fully functional. In contrast, contigs representing members of the CBST family appeared as the most differentially expressed in our RNA-seq analysis, and Southern blot assays failed to show any hybridization to CBTS-homologous sequences in TI 1406, using *NtCBTS-2a* as a hybridization probe. Our collective data presents compelling evidence that a small CBTS gene family (comprised as a minimum of *NtCBTS-2a* and *NtCBTS-*3) is found on the region of chromosome 21 that is deleted in plants possessing the *VAM* locus, but not *va.* The failure to directly assign any CBTS gene to this region, however, is likely attributable to the fact that neither of the two large publically available tobacco genome sequencing initiatives could place CBTS-homologous sequences on any scaffold greater than 2640 bp in size (AWOJ01S331035.1). Thus, it is likely that sequences in the vicinity of the CBTS gene family are recalcitrant to sequencing and/or assembly by the approaches used by both groups. Support for the CBTS gene family residing on chromosome 21 also comes from Southern blotting assays conducted using genomic DNA from the line Red Russian Null E (a line nullisomic for chromosome E, which is the older nomenclature for chromosome 21). Like the TI 1406 Southern blots, no hybridization to *NtCBTS-2a* probes could be detected in Red Russian Null E, providing additional evidence that the CBTS gene family resides on this chromosome (data not shown).

The expression of a *NtCBTS-2a* transgene in TI 1406 under the transcriptional control of either the 35S promoter, or its own native promoter, failed to restore CBT-diol production in these plants. *Z*-abienol was also not detected in any plant (data not shown), despite there being a functional pathway for its synthesis in TI 1406. In tomato, a recent study provided evidence supporting a model in which photosynthesis within the glandular trichomes is largely dedicated toward supplying the energy and reducing power needed to produce secondary metabolites (including terpenoids) specific to that cell type [26]. In addition, Kandra et al. [27] showed that diterpene biosynthesis in tobacco accession TI 1068 is dependent on light, and can be inhibited by the photosynthesis inhibitor DCMU. Because of these observations, the authors of this study proposed that the biosynthesis of leaf surface exudates from the heads of glandular trichomes in tobacco is dependent on energy and/or metabolites provided by the chloroplasts in these cells, a notion that was later supported by comparative ultrastructural analysis of the trichome heads of TI 1068 versus TI 1406 [7]. Our results suggest that a gene(s) other than *NtCBST-2a* that lies within the region of the *VAM* deletion that is not shared with *va* is responsible for the development of functional chloroplasts within the heads of secretory trichomes in tobacco. Our current model identifies 41 genes that reside within the ~ 1 Mbp region exclusively missing in *VAM* mutants, and thus represents the list of candidates for enabling normal chloroplast development within the trichome (though more genes will likely be added as the whole tobacco genome sequence becomes further refined and more complete). Included in this region are several potential transcription factors (Additional file 2: Table S2). Furthermore, although multiple lhcb genes have been shown to be missing in *va* [2], an additional lhcb is located on a scaffold expected to be part of *VAM* and not *va* (Nitab4.5_0007068g0040.1 from Additional file 2: Table S2). The effect that the absence of lhcb genes may have on the *va* or *VAM* phenotypes is unknown, but the loss of a requisite trichome-specific lhcb gene may represent one mechanism whereby chloroplast dysfunction within the trichome head could become manifest. Finally, one of the contigs listed in Table 1 (23580c0g1i1) as being differentially expressed between K326 and TI 1406 (and PCR-validated as being absent in the latter) is annotated as encoding an Early Light-Induced Protein (ELIP). In Arabidopsis, ELIPs have been shown to be integral thylakoid membrane proteins that function to protect leaves from photooxidative stress [28]. Should the ELIP encoded by contig 23580c0g1i1 prove to be exclusively produced in trichomes, it may represent a particularly promising candidate for *VAM*, as the inability to protect trichome chloroplasts from oxidative damage when exposed to high light could explain their ultimate absence/dysfunction within this specific cell type.

## Conclusions

The results presented here provide an in depth characterization of the deletion mutants *va* and *VAM*, two loci of both historical and commercial importance in tobacco. By coupling results obtained from RNA-seq analyses with publically available whole genome sequence information, we were able to develop an initial physical map of the region of chromosome 21 that is deleted in tobacco plants containing the *va* or *VAM* loci. The characterization of select genes within these deletion mutants lead to confirmation of the role of *eiF4E1.S* in facilitating susceptibility to potyvirus infection, as well as the discovery that the small CBTS gene family involved in the biosynthesis of the major class of diterpenes found on the leaf surface of tobacco appears to be missing in plants possessing *VAM.* Finally, although the majority of the chromosomal deletion that defines *VAM* is also deleted in *va*, within the deletion region unique to *VAM* are one or more genes responsible for the production of functional chloroplasts within the trichome head. The results of this study provide the foundation for further investigation of the mechanisms by which viable chloroplasts are maintained within the heads of glandular trichomes.

## Methods

### Plant growth conditions

Tobacco plants for all experiments were grown at room temperature using a 16 h light, 8 h dark cycle. Plants transformed with various *NtCBTS-2a* or vector control constructs were transferred to a greenhouse at approximately the eight-leaf stage and grown to maturity prior to analysis of leaf surface diterpene content. K326 seed was purchased from Gold Leaf Seed Company (Hartsville, SC) and TI 1406 is available from the North Carolina State University Tobacco Germplasm Collection (https://npgsweb.ars-grin.gov/gringlobal/site.aspx?id=25).

### Sequencing and transcriptome assembly

Total RNA was isolated from 100 mg of pooled young leaf tissue of tobacco lines K326, K326-*va* and TI 1406 using the Trizol reagent (Invitrogen, Carlsbad, CA) according to the manufacturer’s protocol. RNA quality was established using a 2100 Bioanalyzer (Agilent Technologies). HiSeq sample preparation was completed using the TruSeq RNA Library Prep Kit v2 (Illumina Inc. San Diego, CA, USA). All three samples were multiplexed on a single lane of an Illumina HiSeq chip for 100 bp paired-end sequencing. For certain applications, the K326 RNA-seq reads deposited in GenBank by Sierro et al. (SRR955772) [2] were also downloaded and utilized.

Processing of raw sequencing reads was performed on all four RNA-seq sets with the Trimmomatic tool (v0.32) [29] using the options: LEADING:13 TRAILING:13 SLIDINGWINDOW:3:21 MINLEN:55. The Trinity de novo assembly package (v2.0.6) was used to assemble a transcriptome using only our processed K326 reads [30, 31]. Contigs under 500 bp in length were removed as well as contigs with under a 10-fold coverage.

### Aligning reads back to transcriptome for deletion discovery

To determine differentially expressed contigs, the processed reads from K326, K326-SRR955772, K326-*va* or TI 1406 were mapped back to the assembled K326 transcriptome using BWA-MEM (v0.7.12) with the options: -c 60,000 [32]. Samtools Idxstats function was used to collect data on alignments.

### PCR and southern blot analysis

PCR amplification of contigs from Table 1 and amplification done to verify and examine scaffolds was performed with primers listed in Additional file 1: Table S1. *Taq* DNA Polymerase (New England BioLabs, Ipswich, MA) was used with thermal cycling conditions consisting of an initial denaturing of 95 °C for 30s; 32 cycles of denaturation at 95 °C for 10s, annealing at 60 °C for 20s, and extension at 68 °C for 1 m and a final cycle of extension at 68 °C for 5 m. Quantitative real-time PCR was conducted as previously described [33].

### Vector construction and plant transformation

Cloning the entire cDNA coding region of *eiF4E1.S* from K326 (corresponding to GenBank accession KF155696) was followed by verification via DNA sequencing. Primers eIF4E_C_F and eIF4E_C_R containing BamHI and SacI sites, respectively, at their 5′ ends were used to amplify the complete open reading frame. PCR products were digested with BamHI and SacI and ligated into plant transformation vector pBI121 that had been digested with the same enzymes (replacing the GUS gene with *eiF4E1.S*) to produce construct 35S:: eiF4E1.S. A full-length *NtCBTS-2a* cDNA and its corresponding promoter were amplified using primers CBTS2aF/CBTS2aR and cembPro_F/cembPro_R, respectively (again placing BamHI and SacI sites on their respective ends). Included in the amplified cDNA were the first 51 codons of the gene which are predicted by ChloroP (www.cbs.dtu.dk/services/ChloroP) to encode the N-terminal transit peptide needed to transport the CBTS enzyme into the chloroplast, the site of diterpene synthesis in plants. The 35S::CBTS-2a construct was generated by replacing the GUS gene in pBI121 with the *NtCBTS-2a* cDNA using BamHI and SacI digestion and ligation as described for 35S::eiF4E1.S. The *NtCBTS-2a* promoter (CEMBpro) was isolated by amplifying a 1812 bp fragment immediately upstream of the ATG start codon. The CEMBpro::GUS construct was generated by replacing the GUS gene in pCAMBIA-1391 using SalI and BamHI sites. CEMBpro::CBTS-2a was made using CEMBpro and CBTS-2a linked at a BamHI site and integrated into pCAMBIA-1390 at SalI and EcoRI sites. Primers mentioned here are provided in Additional file 1: Table S1. All vectors described in this study were subsequently transformed into *Agrobacterium tumefaciens* GV3101 for plant later transformation using the freeze/thaw shock method [34].

### PVY and TEV viral inoculations

Potyvirus strains were maintained on susceptible tobacco cultivars in insect-proof cages. Strain PVY^ZT-5^ was isolated from a tobacco field in Yunnan Province, China and is maintained by the Yunnan Academy of Tobacco Agricultural Sciences. TEV and nectrotic PVY strain PVY^NN^ are maintained at North Carolina State University. Virus inoculum was prepared by macerating systemically infected leaf tissue in phosphate buffer using a mortar and pestle. Approximately 1% (*w*/*v*) of quartz sand (200 mesh) was added to the inoculum and filtered through a 40 mesh Nylon net. Plants were inoculated at approximately the 7–8 leaf stage. Inoculum was applied to two leaves per plant using a high-pressure spray gun. Plants were evaluated either 14 or 21 days after inoculation.

### GC-MS analysis

Trichome exudate analysis was performed using GC-MS with minor changes as described by Vontimitta et al. [34]. Briefly, ten 1.5 cm leaf punches per plant were collected and pooled. Samples were immediately placed on ice and remained there until analysis. Leaf surface exudates were collected by washing the leaves twice with CH_2_Cl_2_, adding Na_2_SO_4_, and incubating overnight. An Agilent HP 6890 GC-FID (Santa Clara, CA) was used for gas chromatographic analysis with a helium carrier.

## Additional files

Additional file 1: **Table S1.** Primer sequences. (DOCX 551 kb)

Additional file 2: **Table S2.** Annotations of genes within scaffolds expected to be part of VAM. (XLSX 10 kb)

Additional file 3: **Table S3.** PVY infection of TN86 plants. Table showing results of phenotypic observation and immunochemical analysis of 14 T0 35S::eiF4E1.S (OX) and 10 T0 vector control (VC) plants in the TN86 background. Positive (+) scoring of PVY symptoms was based on observation of mosaic patterning of leaves. (XLSX 13 kb)

Additional file 4: **Figure S1.** Typical examples of potyvirus infection of TI 1406 and K326-*va* plants transformed with 35S::eiF4E1.S construct or vector control (VC). Pictures were taken 14 days post-infection with PVY^NN^ or TEV. (DOCX 54 kb)

## Abbreviations

BMVSE: ß-methylvaleric acid-containing sucrose esters
CBT: cembratriene
CBTS: cembratrienol synthase
DH: doubled haploid
eiF4E: eukaryotic translational initiation factor 4E
ELIP: early light-induced protein
GGPP: geranylgeranyl diphosphate
lhcb: light-harvesting chlorophyll a/b binding protein
PVY: potato virus Y
TEV: tobacco etch virus
TI: tobacco introduction
TVMV: tobacco vein mottling virus
VAM: Virgin A Mutant
