# Supplementary figures and images for: Characterization of Nicotiana tabacum genotypes possessing deletion mutations that affect potyvirus resistance and the production of trichome exudates
Source: BMC Genomics. 2018 Jun 20;19:484. doi: 10.1186/s12864-018-4839-y (PMC6011258; doi:10.1186/s12864-018-4839-y)

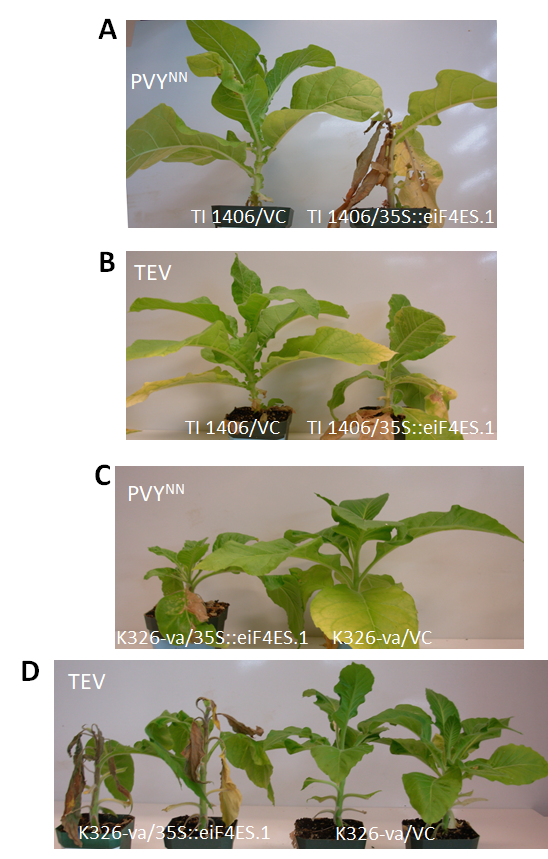

Supplement: Supplementary file 1 — Table S1. Primer sequences. (DOCX 551 kb) [file 12864_2018_4839_MOESM1_ESM.docx]

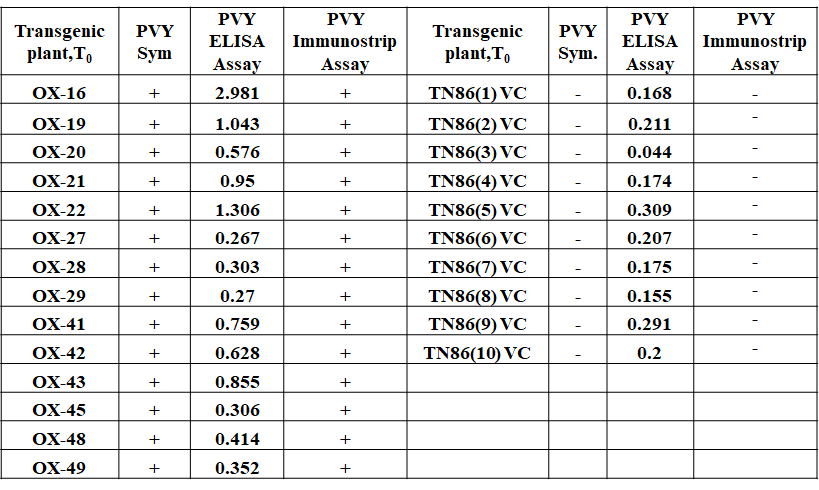

Supplement: Supplementary file 4 — Figure S1. Typical examples of potyvirus infection of TI 1406 and K326-va plants transformed with 35S::eiF4E1.S construct or vector control (VC). Pictures were taken 14 days post-infection with PVYNN or TEV. (DOCX 54 kb) [file 12864_2018_4839_MOESM4_ESM.docx]
